# QuickStep-Cloning: a sequence-independent, ligation-free method for rapid construction of recombinant plasmids

**DOI:** 10.1186/s13036-015-0010-3

**Published:** 2015-09-18

**Authors:** Pawel Jajesniak, Tuck Seng Wong

**Affiliations:** ChELSI Institute and Advanced Biomanufacturing Centre, Department of Chemical and Biological Engineering, University of Sheffield, Mappin Street, Sheffield, S1 3JD UK

**Keywords:** Molecular cloning, Gene cloning, Megaprimer, Recombinant DNA, Recombinant plasmid, Protein engineering, Directed evolution, Synthetic biology, Metabolic engineering

## Abstract

**Background:**

Molecular cloning is an essential step in biological engineering. Methods involving megaprimer-based PCR of a whole plasmid are promising alternatives to the traditional restriction-ligation-based molecular cloning. Their widespread use, however, is hampered by some of their inherent characteristics, *e.g.*, linear amplification, use of self-annealing megaprimers and difficulty with performing point insertion of DNA. These limitations result in low product yield and reduced flexibility in the design of a genetic construct.

**Result:**

Here, we present a novel technique of directional cloning, which overcomes these problems yet retaining the simplicity of whole-plasmid amplification. QuickStep-Cloning utilizes asymmetric PCRs to create a megaprimer pair with 3′-overhangs, and hence, facilitates the subsequent exponential whole-plasmid amplification. QuickStep-Cloning generates nicked-circular plasmids, thereby permitting direct bacterial transformation without DNA ligation. It allows DNA fragment integration into any plasmid at any position, in an efficient, time- and cost-effective manner, without tedious intermediate DNA gel purification, modified oligonucleotides, specialty enzymes and ultra-competent cells. The method is compatible with competent *E. coli* cells prepared using the conventional calcium chloride method.

**Conclusion:**

QuickStep-Cloning expands the versatility of megaprimer-based cloning. It is an excellent addition to the cloning toolbox, for the benefit of protein engineers, metabolic engineers and synthetic biologists.

**Electronic supplementary material:**

The online version of this article (doi:10.1186/s13036-015-0010-3) contains supplementary material, which is available to authorized users.

## Background

Gene cloning is an indispensable molecular biology technique that, since its first introduction, has been central to the development of genetic engineering and, consequently, the entire field of life sciences. Despite its widespread use, the traditional, restriction-ligation-based cloning protocol suffers from major problems, including, but not limited to: (i) low efficiency, (ii) dependency on the availability of unique restriction sites in a cloning vector and in the gene of interest, and (iii) time-consuming and labour-intensive process. In recent years, many novel approaches to molecular cloning have been proposed to expedite the procedure, enhance cloning efficiency and bypass the requirement of restriction sites [1, 2]. Homologous recombination [3, 4], incorporation of phosphorothioate oligonucleotides [5] and use of zinc finger nucleases [6] are only a few examples of different strategies utilized for this purpose.

Among the reported approaches to DNA cloning, methods involving megaprimer-based PCR of a whole plasmid, *e.g.*, restriction site-free cloning [7], restriction-free (RF) cloning [8], overlap extension PCR cloning [9] and MEGAWHOP cloning [10], have attracted a significant interest among the scientific community. These methods were inspired by the hugely popular and easy-to-use QuikChange™ (Agilent) protocol for site-directed mutagenesis [11]. Despite their indisputable potential, megaprimer-based methods are inherited with several drawbacks that compromise their overall efficiency: (i) linear amplification of the recipient vector, (ii) use of a completely complementary megaprimer pair, (iii) difficulty with performing point insertion of DNA, (iv) random mutations introduced by the DNA polymerase of choice during whole-plasmid amplification, and (v) poor amplification of GC-rich DNA fragments. The listed drawbacks significantly decrease the overall efficiency of the cloning method and, consequently, necessitate the use of enzyme-based DNA ligation and time-consuming optimization of PCR conditions to achieve a sufficient number of transformants containing recombinant DNA of interest. Four recently proposed cloning methods, asymmetric bridge PCR with intramolecular homologous recombination [ABI-REC, [12]], recombination-assisted megaprimer (RAM) cloning [13], exponential megaprimer PCR (EMP) cloning [14], and inverse fusion PCR cloning [IFPC, [15]], have been reported to achieve exponential amplification via incorporating additional oligonucleotides into megaprimer PCR. In all cases, however, the amplification results in generation of linearized plasmids instead of the more desirable circular DNA. ABI-REC and RAM are homologous recombination-dependent methods, relying on transformation of linearized plasmids and their repair *in vivo*, which usually provides significantly less transformants than transformation of nicked or intact plasmids. On the other hand, EMP and IFPC cloning protocols require phosphorylation and ligation to circularize the amplification products.

Here, we report QuickStep-Cloning, a novel method that builds upon the simplicity of QuikChange™. Not only it addresses major drawbacks of traditional DNA cloning, the method also circumvents the aforementioned problems of the existing megaprimer-based cloning methods, including the problem of linear amplification and self-annealing megaprimers.

## Results and discussion

### QuickStep-Cloning: Principle and molecular mechanism

**Fig. 1:**
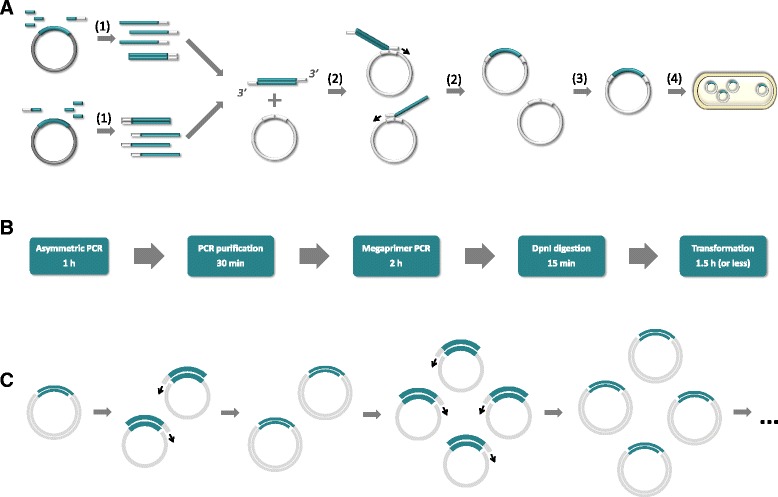
Overview of QuickStep-Cloning: **a** A schematic diagram presenting individual stages involved in the proposed method: (1) two parallel asymmetric PCRs of DNA of interest and PCR purification, (2) megaprimer-based PCR, (3) DpnI digestion, and (4) bacterial transformation. **b** Exemplary workflow for 1 kb insert and 7 kb cloning vector (exact duration of the asymmetric PCR depends on the length of cloned DNA fragment and the duration of megaprimer PCR is related to the size of the cloning vector). **c** Outline of exponential amplification taking place during QuickStep-Cloning – megaprimers anneal themselves to the product of linear amplification and are extended by polymerase, producing further copies of the two single-stranded templates in an exponential manner. It should be noted that for the given mechanism, exponential amplification occurs in parallel with the linear process

In QuickStep-Cloning, DNA fragment of interest is amplified in two parallel asymmetric PCRs [16], during which regions complementary to the integration site on the recipient plasmid are added to both ends of the amplified DNA fragment (Fig. 1). The products of the two asymmetric PCRs are purified, mixed and used as megaprimers for the consecutive PCR. In contrary to the traditional megaprimer-based PCR of a whole plasmid, the megaprimer pair in QuickStep-Cloning contains 3′-overhangs (instead of blunt ends) allowing it to anneal to the recipient plasmid even when the two megaprimers self-anneal. Megaprimers designed in this way facilitate an exponential amplification, which results in production of nicked-circular plasmids. After a short incubation with DpnI to remove methylated/hemimethylated recipient plasmids that do not contain gene of interest, the product of the megaprimer PCR can be directly used for transformation. For a 1-kb gene and a 7-kb recipient plasmid, for instance, the entire workflow can be completed in less than 6 h (Fig. 1).

### Primer design for QuickStep-Cloning

**Fig. 2:**
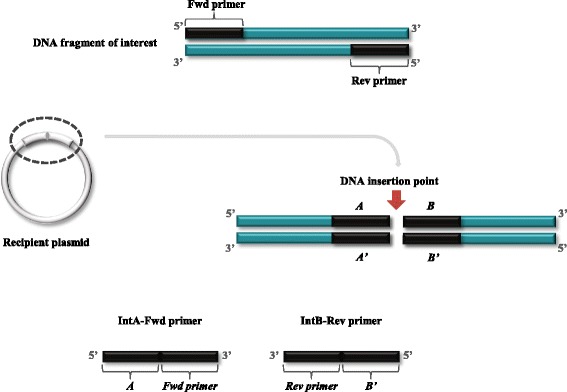
Outline of primer design for QuickStep-Cloning. The sequences of *Fwd* and *Rev* primers are derived from the target gene sequence. *IntA-Fwd* and *IntB-Rev* are chimeric primers, carrying sequence of integration site (5′-portion) and target gene sequence (3′-portion)

QuickStep-Cloning allows point integration of a gene at any position of any recipient plasmid. This is achieved through judicious design of the four primers (denoted as *Fwd*, *Rev*, *IntA-Fwd* and *IntB-Rev*, Fig. 2) that are used in the two parallel asymmetric PCRs. *Fwd* and *Rev* are short primers derived from the target gene sequence only. *IntA-Fwd* and *IntB-Rev*, are chimeric primers, carrying both the sequence upstream or downstream to the integration site and the target gene sequence. Asymmetric PCR with unbalanced concentration of *Fwd* (500 nM) and *IntB-Rev* (10 nM) primers results in sense strands with integration sequence at 3′-termini. Likewise in another asymmetric PCR using 10 nM of *IntA-Fwd* and 500 nM of *Rev*, antisense strands with integration sequence at 3′-termini are produced. When both strands from the two asymmetric PCRs are purified and mixed, megaprimer pairs with 3′-overhangs are produced for use in the subsequent megaprimer PCR step.

### Demonstration of QuickStep-Cloning

To investigate the efficiency of the proposed design, QuickStep-Cloning was utilized to transfer a DNA fragment from pEGFP vector (containing ampicillin resistance gene; Additional file 1: Figure S1) into pET24a-HLTEV-p53 plasmid (containing kanamycin resistance gene). The primers were designed to perform a point insertion of *egfp* gene just before the p53 open reading frame (Additional file 1: Figure S2), producing kanamycin-resistant transformants capable of EGFP expression. After 30 cycles of asymmetric PCR and 25 cycles of megaprimer PCR, *E. coli* strains DH5α and C41 (DE3) were transformed with 5 μl of DpnI-digested PCR product and plated on agar plates supplemented with ampicillin or kanamycin and IPTG (Table 1). EGFP-expressing colonies were easily discernible for C41 (DE3) grown on IPTG-supplemented plates (Additional file 1: Figure S3). The accuracy of visual inspection of the transformants has also been further verified, by selecting randomly five EGFP-negative colonies and growing them at 30 °C for 24 h in TB-based auto-induction media – no fluorescence was detected for all five clones (Additional file 1: Figure S4). Worthy of note, there is no need to first remove *p53* gene that is pre-cloned into the recipient vector, highlighting a useful feature of QuickStep-Cloning. Further, the product of QuickStep-Cloning can be directly transformed into an expression strain [such as C41 (DE3)] for protein expression, bypassing the intermediate cloning strain (DH5α). Plasmids from ten randomly selected EGFP-expressing colonies were sequenced and the presence of DNA insert in the recombinant pET24a-HLTEV-p53 has been confirmed for all 10 clones. Worthy of note, no undesired mutation was found within the *egfp* gene in any of the 10 clones. In 9 cases, *egfp* gene was inserted at desired position in the right orientation. One plasmid contained two copies of *egfp* gene separated by a 28 bp sequence, containing partial sequence of *IntA-EGFP-Fwd* and *IntB-EGFP-Rev* primers. This construct is, most likely, a result of megaprimer-dimer formation during whole plasmid amplification. Concurrently, plasmids from five EGFP-negative colonies were sequenced – one clone contained no insert and the remaining four carried unwanted mutations in *egfp* gene. Three of them contained single base substitutions. One contained three single base substitutions and one 3 bp deletion, all present in the region where primers *EGFP-Fwd* or *IntA-EGFP-Fwd* bind.

**Table 1:** Results of *egfp* cloning experiment

Strain, Selection plate	QuickStep-Cloning	RF Cloning	Transformation efficiency [cfu/μg]
DH5α, Ampicillin	0	0	3.8 · 10^4^
DH5α, Kanamycin	476	35	3.8 · 10^4^
C41(DE3), Kan + IPTG	618(575)	160(7)	4.2 · 10^6^

Colony counts for *E. coli* strains DH5α and C41 (DE3) transformed with the products of RF cloning and of QuickStep-Cloning and plated on agar plates supplemented with: (i) 100 μg/ml ampicillin, (ii) 50 μg/ml kanamycin, and (iii) 50 μg/ml kanamycin and 1 mM IPTG. Transformation efficiency was determined based on concurrent transformation of 1 ng intact pET24a-HLTEV-p53 plasmid. Numbers in the brackets denote EGFP-expressing colonies, as determined by visual inspection using UV transilluminator. Lack of colonies observed on ampicillin-supplemented agar plates indicated that the final PCR mixture produced via QuickStep-Cloning, used directly for bacterial transformation, did not contain significant amount of donor plasmid

### Optimizing QuickStep-Cloning

**Fig. 3:**
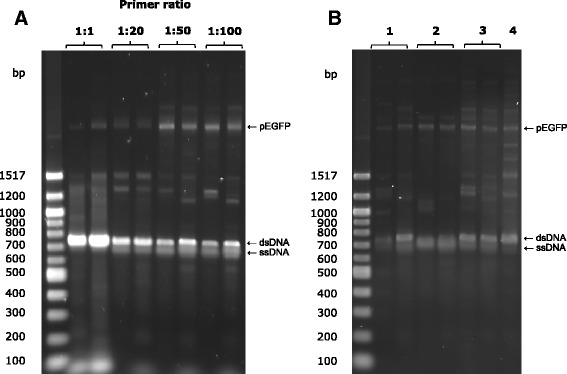
Investigation and optimization of asymmetric PCR stage of QuickStep-Cloning. **a** Yield of two parallel asymmetric PCRs for different primer ratios (represented by two separate rows for each ratio). **b** Identification of single stranded product of asymmetric PCR stage – (1) individual products of two parallel asymmetric PCRs, (2) both products after 2 min denaturation at 94 °C, (3) renatured products, and (4) products of two parallel asymmetric PCRs after being mixed together. In all gel pictures, the appearance of low and high molecular weight bands could be attributed to non-specific binding of primers, commonly seen in regular PCRs

**Fig 4:**
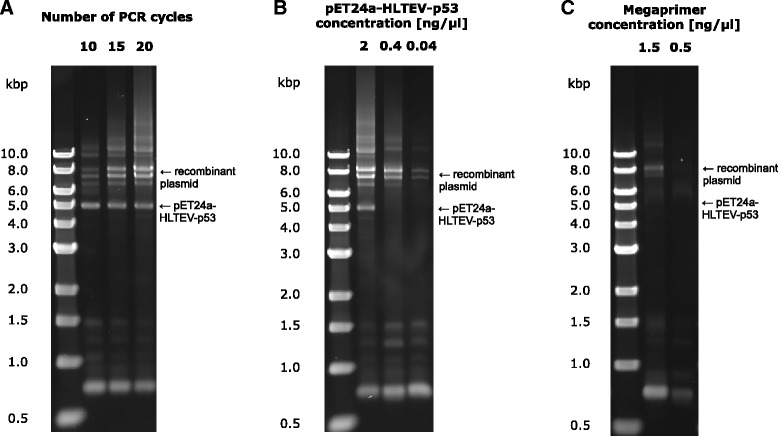
Optimization of megaprimer PCR stage of QuickStep-Cloning. **a** Yield of megaprimer PCR for varying number of PCR cycles. **b** Yield of megaprimer PCR for different concentrations of recipient plasmid. **c** Yield of megaprimer PCR for different concentrations of megaprimer. In all gel pictures, the appearance of low and high molecular weight bands could be attributed to non-specific binding of primers, commonly seen in regular PCRs

The success of QuickStep-Cloning is attributed to our ability to produce (1) ssDNA in sufficient quantity in the two asymmetric PCRs, and (2) high yield of megaprimer PCR. To address the former, the primer ratio in asymmetric PCRs (*i.e.*, the ratio of *Fwd-IntA* to *Rev* and the ratio of *Rev-IntB* to *Fwd*) was optimized (Fig. 3). At a ratio of 1:1, the PCR product was predominantly dsDNA, which was excellently stained by Diamond Nucleic Acid Dye from Promega (Fig. 3a). At ratios of 1:20, 1:50 and 1:100, a lower gel band corresponding to ssDNA product started to appear. ssDNA migrates faster in agarose gel compared to its dsDNA counterpart and is less efficiently stained by fluorescent dye. A series of denaturation and annealing experiments were conducted (Fig. 3b), confirming the identity of these lower gel bands. The effect of primer ratio on the efficiency of the proposed cloning method has further been investigated by analyzing subsequent whole plasmid amplification (Additional file 1: Figure S5) and, based on the results obtained, 1:50 ratio was concluded to be the most optimal. To obtain good product yield in megaprimer PCR, three parameters were carefully optimized, namely number of PCR cycles, concentration of recipient plasmid and megaprimer concentration (Fig. 4).

### Comparison to restriction-free (RF) cloning

To demonstrate the superior performance of QuickStep-Cloning, restriction-free (RF) cloning was carried out in parallel for comparison, using identical reaction conditions and primer design. QuickStep-Cloning provided much higher number of transformants - 93 % of which contained recombinant plasmid (Table 1). RF cloning provided 160 transformants, only 4 % of which displayed fluorescence. Five out of only seven EGFP-expressing colonies obtained via RF cloning were used for subsequent sequencing – four plasmids contained the desired insert at the right orientation. One of the plasmids included not only a single mutation within the *egfp* gene but also an additional long (>100 bp) DNA fragment located between *egfp* and *p53* genes, containing partial sequence of *IntA-EGFP-Fwd* and *IntB-EGFP-Rev* primers. Poor efficiency of RF cloning might be attributed to lack of DNA ligation and, most importantly, inherent difficulties with point insertion of DNA, characteristic to many cloning methods relying on megaprimer-based PCR of a whole plasmid. Important to note, in the case of RF cloning it is advised to have a distance of 50 to several hundred base pairs between the two annealing sites on the recipient plasmid [9], necessitating removal of a short DNA sequence between both annealing sites during cloning. Sequencing 5 plasmids isolated from EGFP-negative colonies showed that three of them contained a relatively short (~30 bp) DNA insert instead of a desired *egfp* gene, plausibly a result of mispriming and primer-dimer formation. The remaining two did not return readable sequences.

### General applicability of QuickStep-Cloning

QuickStep-Cloning is not limited to transfer of genes between two plasmids carrying distinct selection markers (in the case of *egfp* cloning, the gene was transferred from Amp^r^-pEGFP to Kan^r^-pET24a-HLTEV-p53). To investigate the robustness of the developed protocol, QuickStep-Cloning method was applied to another system. The *rfp* gene from Kan^r^-pBbA8k-RFP (containing *rfp* gene, under the control of arabinose-inducible promoter; Additional file 1: Figure S6) was successfully cloned into Kan^r^-pET24a-HLTEV-p53 using QuickStep-Cloning. The only differences in the protocol from *egfp* cloning experiment have been the use of a new set of four primers, designed following general guidelines presented in this paper, and the corresponding annealing temperatures. Without any further optimization, QuickStep-Cloning again exhibited superior performance in comparison to RF cloning (Table 2), providing 418 colonies, 97 % of which expressed RFP. The accuracy of visual inspection of agar plates has been confirmed by further expression studies (Additional file 1: Figure S8 and S9). Sequencing has shown that out of five investigated RFP-expressing transformants, all five of them contained pET24a vector with *rfp* insert. Only one clone contained unwanted mutation, namely, a 5-bp deletion downstream of *egfp* gene (*i.e.*, at the vector integration site). Interestingly, plasmids from five out of only 15 observed RFP-negative colonies have also been scrutinized and all of them had short deletions at or close to start codon of *rfp* gene, where primers *RFP-Fwd* or *IntA-RFP-Fwd* bind. The localization of these unwanted mutations within the primer-binding region is unlikely to be purely coincidental. It is hypothesized that these artifacts could be derived from the impurities (*e.g.*, deletion products) present in the synthetic oligonucleotides. As the occurrence of occasional mutations, especially deletions, is a widely-known shortcoming of long, desalted primers, it is envisaged that the use of HPLC-purified primers can further improve the already exceptionally high efficiency of QuickStep-Cloning.

**Table 2:** Results of *rfp* cloning experiment

Strain, Selection plate	QuickStep-Cloning	RF Cloning	Transformation efficiency [cfu/μg]
DH5α, Kanamycin	334	26	3.8 · 10^4^
C41(DE3), Kan + IPTG	418(404)	113(103)	4.2 · 10^6^

Colony counts for *E. coli* strains DH5α and C41 (DE3) transformed with the products of RF cloning and of QuickStep-Cloning and plated on agar plates supplemented with: (i) 50 μg/ml kanamycin and (ii) 50 μg/ml kanamycin and 1 mM IPTG. Transformation efficiency was determined based on concurrent transformation of 1 ng intact pET24a-HLTEV-p53 plasmid. Numbers in the brackets denote RFP-expressing colonies, as determined by visual inspection of the plates

### Comparison to other cloning methods

In order to highlight the novelty of and the benefits offered by QuickStep-Cloning, the proposed method was compared with four recently reported strategies of exponential megaprimer-based cloning (ABI-REC, RAM cloning, EMP cloning and IFPC); the results of this comparison are summarized in Table 3. QuickStep-Cloning is one of the first cloning methods fully optimized for use with the recently developed Q5 High-Fidelity DNA Polymerase (New England Biolabs), which is characterized by its ultra-low error rate (200× higher fidelity than *Taq* polymerase and approximately 2× higher fidelity than the widely-used Phusion polymerase), very high speed of DNA replication (6 kb/min) and superior performance for a broad range of amplicons, including DNA with a high GC content. The presented method was demonstrated to be suitable for direct transformation of not only widely used *E. coli* cloning strain (DH5α) but also a common expression strain, C41 (DE3). Worthy of note, the two distinct experiments utilizing QuickStep-Cloning (cloning of *egfp* and *rfp* genes) provided hundreds transformants (Tables 1 and 2), despite the use of a relatively simple transformation method (allowing for transformation efficiencies in the range of just 10^4^-10^6^ cfu per μg of intact plasmid). In comparison, many of the previously-reported methods were investigated based solely on highly-efficient transformation protocols. For example, overlap extension PCR cloning, a method utilizing the principles of RF cloning, was reported to produce up to 600 colonies from small aliquots of final PCR mixture [9]. However, the chemically competent *E. coli* cells used in that study had been prepared via Inoue method, a time-consuming protocol which allows to achieve transformation efficiencies exceeding 10^9^ cfu/μg [17]. In stark contrast to the other four recently reported methods, QuickStep-Cloning does not rely on either undesirable *in vivo* homologous recombination or enzymatic phosphorylation-ligation process. The whole cloning procedure requires only one PCR purification step, whereas both RAM cloning and IFPC involve time-consuming gel purification. Based on a rough estimate of time needed to integrate a 1 kb DNA fragment into a 7 kb plasmid using the six different megaprimer-based cloning methods, QuickStep-Cloning emerges as an unquestionable winner when it comes to overall cloning time. Most importantly, its cloning efficiency compares favourably to the values reported for the remaining five methods. The only drawback of QuickStep-Cloning is its requirement of four distinct primers (difference of one additional short primer in comparison to the other exponential cloning methods). Even though there is a chance of accidental DNA misinsertion (no such cases have yet been identified throughout our study), *Fwd* and *Rev* primers can be useful in colony PCR for quick identification of plasmids with gene insert. Of course, the use of primers complementary to vector regions flanking the insertion site is most appropriate for identifying clones with gene inserted at desired location.

**Table 3:** A comparison of QuickStep-Cloning to other recently reported megaprimer-based cloning methods. Desirable features are highlighted in bold to facilitate comparison

Cloning method	QuickStep-Cloning	RF	ABI-REC	RAM	EMP	IFPC
Cloning strategy	**Megaprimer**	**Megaprimer**	**Megaprimer**	**Megaprimer**	**Megaprimer**	**Megaprimer**
Amplification mode	**Exponential**	Linear	**Exponential**	**Exponential**	**Exponential**	**Exponential**
Transformed product	Nicked-circular plasmid (2 nicks per plasmid)	Nicked-circular plasmid (2 nicks per plasmid)	Linear DNA	Linear DNA	**Closed-circular plasmid**	**Closed-circular plasmid**
*E. coli* cells used	**Chemically competent DH5α and C41 (DE3)**	Electrocompetent TG1	Chemically competent DH5α	Strain type not reported	Chemically competent DH5α	Chemically competent TOP10
*In vivo* homologous recombination	**No**	**No**	Yes	Yes	**No**	**No**
Enzymatic phosphorylation-ligation	**No**	**No**	**No**	**No**	Yes	Yes
Number of primers required	4	**2**	3	3	3	3
Gel purification	**No**	**No**	**No**	1×	**No**	Strongly recommended
PCR purification	1×	1×	**No**	**No**	2×	No
Estimated cloning time^a^	**5 h 15 min**	14 h	7 h 45 min	7 h 45 min	7 h 15 min	6 h 30 min
Reported cloning efficiency^b^	**93–97 %**	**~90 %** ^c^	**93–100 %**	**75–94 %**	**10–100 %**	**~90 %**
*Reference*	*-*	[8]	[12]	[13]	[14]	[15]

^a^As estimated for cloning 1 kb DNA fragment into 7 kb plasmid according to originally reported protocol (for more information see Additional file 1)

^b^ Judging by the percentages reported, all methods are capable of delivering similar efficiency. Worthy of note, these numbers are dependent on the approaches used by the authors to evaluate cloning efficiency

^c^As reported in the original paper [8]. Ulrich et al. [14] and Mathieu et al. [13] demonstrate, respectively, 27 and 16 % efficiency for RF cloning

Based on the presented facts, QuickStep-Cloning fares exceptionally well in comparison to other, previously-reported megaprimer-based cloning methods. However, what about more popular cloning methods such Gibson Assembly cloning [18, 19] or Ligation Independent Cloning (LIC) [20]? Both of these methods are often advertised as being able to achieve full cloning in less than an hour and less than 3 h, respectively, appearing to be much faster when compared to 6 h duration time provided for QuickStep-Cloning. Surprisingly, these general estimates usually not only neglect the time needed for bacterial transformation but also assume that two DNA fragments to be joined already contain complementary terminal regions and that recipient plasmid is already linearized. The last point is particularly salient in the case of any general cloning experiment utilizing Gibson Assembly or LIC, as recipient plasmid has to be linearized, most often than not, with either restriction enzymes or inverse PCR. Use of restriction enzymes for this purpose introduces a host of problems inherent to the traditional, restriction-ligation-based cloning protocol, such as dependency on the availability of unique restriction sites in a cloning vector. Application of inverse PCR allows for sequence-independent cloning, however, it provides some of the drawbacks associated with megaprimer-based cloning (*e.g.*, reliance on error-prone polymerase of choice and necessity of careful primer design). If Gibson assembly was to be used together with inverse PCR to clone 1 kb DNA fragment into 7 kb expression vector (analogous to the proof-of-concept *egfp* cloning experiment presented in here), according to our conservative estimates, about 3 h would be needed to perform the inverse PCR and subsequent DpnI digestion (to remove any traces of parental vector) and plasmid purification. Adding to this the time needed to perform enzymatic assembly and bacterial transformation, the total time of performing cloning via Gibson Assembly appears to be comparable to QuickStep-Cloning. Worthy of note, recipient plasmid linearization and amplification of DNA insert combined with introduction of complementary overhangs require design of the same number of primers as QuickStep-Cloning.Taking into account the cost of enzymatic reaction components (T5 exonuclease, Taq ligase, suitable polymerase and appropriate buffer sustaining simultaneous activity of all three enzymes) and the need of synthesizing four different primers, Gibson Assembly cloning seems to be more costly and resource-intensive than QuickStep-Cloning. What is more, use of highly-competent bacterial strains for Gibson Assembly is highly recommended. Without shadow of a doubt, Gibson Assembly remains a powerful and highly versatile molecular-biology tool, which involves a broad range of applications including, but not limited to, multiple-fragment assembly and molecular cloning coupled to simultaneous deletion of a DNA fragment. The same argument applies equally well to Ligation Independent Cloning. In our opinion, however, for certain applications such as point insertion of long DNA stretches into a cloning vector, QuickStep-Cloning provides an attractive alternative to even the most popular and established cloning methods.

## Conclusions

Based on the presented experimental results, it can be claimed that QuickStep-Cloning is a rapid and highly efficient method of molecular cloning. A DNA fragment of interest can be inserted into any position on the recipient vector and fully cloned in less than 6 h, without the need of DNA ligation and with only one simple PCR purification step. The usefulness of QuickStep-Cloning is certainly not limited to standard cloning experiments, involving transfer of a gene sequence from a donor vector to a recipient plasmid. The developed method could be especially useful for protein tagging or, potentially, cloning DNA fragments directly from genomic DNA. We envisage that QuickStep-Cloning would find its applications in the developing fields of protein engineering, metabolic engineering and synthetic biology.

## Methods

### Materials

All enzymes, deoxyribonucleotides and DNA ladders were purchased from New England Biolabs (Ipswich, USA).

### Primers

Primers used in this study were synthesized by Eurofins Genomics (Ebersberg, Germany). Melting temperatures of oligonucleotides were determined using the New England Biolabs T_m_ Calculator (https://www.neb.com/tools-and-resources/interactive-tools/tm-calculator). Four primers were used in *egfp* cloning experiment: *EGFP-Fwd* (5′- ATGGTGAGCAAGGGCGAG-3′, 18 bp), *IntA-EGFP-Fwd* (5′- CGAAAACCTGTACTTCCAGGGTGGATCCATGGTGAGCAAGGGCGAG-3x′, 46 bp), *EGFP-Rev* (5′-TTACTTGTACAGCTCGTCCATG-3′, 22 bp) and *IntB-EGFP-Rev* (5′- CTAGGATCTGACTGCGGCTCCTCCATTTACTTGTACAGCTCGTCCATG-3′, 48 bp). Underlined parts of *IntA-Fwd* and *IntB-Rev* are identical to *Fwd* and *Rev* primers, respectively, and the remaining parts correspond to the two megaprimer annealing sites flanking DNA insertion point present in pET24a-HLTEV-p53. Similarly, the following four primers were used for *rfp* cloning experiment: *RFP-Fwd* (5′-ATGGCGAGTAGCGAAGACG-3′, 19 bp), *IntA-RFP-Fwd* (5′-CGAAAACCTGTACTTCCAGGGTGGATCCATGGCGAGTAGCGAAGACG-3′, 47 bp), *RFP-Rev* (5′-TTAAGCACCGGTGGAGTGACG-3′, 21 bp) and *IntB-RFP-Rev* (5′- CTAGGATCTGACTGCGGCTCCTCCATTTAAGCACCGGTGGAGTGACG-3′, 47 bp).

### QuickStep-Cloning

To transfer *egfp* gene from pEGFP (Clontech Laboratories, Mountain View, USA) into pET24a-HLTEV-p53 plasmid, two asymmetric PCRs were carried out in parallel. Asymmetric PCR mixture I (50 μl) contained 1× Q5 Reaction Buffer, 200 μM of each dNTP, 500 nM *EGFP-Fwd* primer, 10 nM *IntB-EGFP-Rev* primer, 0.2 ng pEFGP, and 1 U Q5 High-Fidelity DNA Polymerase. Asymmetric PCR mixture II (50 μl) contained 1× Q5 Reaction Buffer, 200 μM of each dNTP, 10 nM *IntA-EGFP-Fwd* primer, 500 nM *EGFP-Rev* primer, 0.2 ng pEFGP and 1 U Q5 High-Fidelity DNA Polymerase. Both mixtures were thermocycled using the following conditions: (i) 30 s initial denaturation at 98 °C and (ii) 30 cycles of 7 s denaturation at 98 °C, 20 s annealing at 65 °C and 30 s extension at 72 °C. The two PCR products were purified using QIAquick PCR Purification Kit (Qiagen, Hilden, Germany) and their DNA concentration was determined using NanoDrop 2000 (Thermo Scientific, Wilmington, USA). For megaprimer PCR, the mixture (50 μl) contained 1× Q5 Reaction Buffer, 200 μM of each dNTP, 200 ng of purified asymmetric PCR product I, 200 ng of purified asymmetric PCR product II, 20 ng pET24a-HLTEV-p53 and 1 U Q5 High-Fidelity DNA Polymerase. The mixture was thermocycled according to the following program: (i) 30 s initial denaturation at 98 °C, (ii) 25 cycles of 10 s denaturation at 98 °C, 4 min annealing and extension at 72 °C, and (iii) 2 min final extension at 72 °C. Forty units of DpnI were subsequently added to the PCR mixture and incubated at 37 °C for 15 min to remove the parental pET24a-HLTEV-p53 plasmids. To clone *rfp* gene from pBbA8k-RFP, purchased from Addgene (plasmid #35273), into pET24a-HLTEV-p53 plasmid, the same protocol was followed, using a dedicated primer set (*RFP-Fwd*, *IntA-RFP-Fwd*, *RFP-Rev* and *IntB-RFP-Rev*) and corresponding annealing temperature of 68 °C (provided by New England Biolabs T_m_ Calculator) for the two asymmetric PCRs.

### Restriction-free (RF) cloning

PCR mixture (50 μl) containing 1× Q5 Reaction Buffer, 200 μM of each dNTP, 500 nM *IntA-Fwd* primer, 500 nM *IntB-Rev* primer, 0.2 ng pEFGP, and 1 U Q5 High-Fidelity DNA Polymerase was thermocycled using the same program as asymmetric PCR in QuickStep-Cloning: (i) 30 s initial denaturation at 98 °C and (ii) 30 cycles of 7 s denaturation at 98 °C, 20 s annealing at 65 °C and 30 s extension at 72 °C. The PCR product was purified using QIAquick PCR Purification Kit and its DNA concentrations was determined using NanoDrop 2000. Megaprimer PCR mixture (50 μl) containing 1× Q5 Reaction Buffer, 200 nM of each dNTP, 400 ng purified PCR product, 20 ng pET24a-HLTEV-p53 and 1 U Q5 High-Fidelity DNA Polymerase was thermocycled in the same conditions as QuickStep-Cloning megaprimer PCR: (i) 30 s initial denaturation at 98 °C, (ii) 25 cycles of 10 s denaturation at 98 °C, 4 min annealing and extension at 72 °C, and (iii) 2 min final extension at 72 °C. Forty units of DpnI were added to the PCR mixture and incubated at 37 °C for 15 min to remove the parental pET24a-HLTEV-p53 plasmids. To clone *rfp* gene from pBbA8k-RFP into pET24a-HLTEV-p53 plasmid, the same protocol was followed, using a dedicated primer set (*RFP-Fwd*, *IntA-RFP-Fwd*, *RFP-Rev* and *IntB-RFP-Rev*) and corresponding annealing temperature of 68 °C for the first PCR.

### DNA gel electrophoresis

PCR products were analyzed using either 0.7 % or 1.5 % agarose gel. DNA was stained using Diamond Nucleic Acid Dye (Promega, Madison, USA). DNA ladders used were Quick-Load 1 kb DNA Ladder and Quick-Load 100 bp DNA Ladder.

### Transformation and clone analysis

*E. coli* DH5α and C41 (DE3) were transformed with 5 μl of DpnI-digested products of QuickStep-Cloning or RF cloning, using a standard chemical transformation protocol [21]. Concurrently, the two bacterial strains were transformed with 1 μl of 1 ng/μl intact pET24a-HLTEV-p53 to estimate transformation efficiency. Transformed bacteria were plated on TYE agar plates (10 g/l tryptone, 5 g/l yeast extract, 8 g/l sodium chloride and 15 g/l agar) supplemented with: (i) 100 μg/ml ampicillin, (ii) 50 μg/ml kanamycin, and (iii) 50 μg/ml kanamycin and 1 mM IPTG. The plates were incubated overnight at 37 °C and for further 12 h at 30 °C. The number of EGFP-expressing colonies was determined by visual inspection using UV transilluminator. Five EGFP-negative colonies, together with one EGFP-expressing colony and one colony containing original pET24a-HLTEV-p53 were used to inoculate separate 5 ml aliquots of TB-based auto-induction media (12 g/l tryptone, 24 g/l yeast extract, 3.3 g/l (NH_4_)_2_SO_4_, 6.8 g/l KH_2_PO_4_, 7.1 g/l Na_2_HPO_4_, 0.5 g/l glucose, 2.1 g/l α-Lactose monohydrate and 0.31 g/l MgSO_4_ · 7H_2_O). After 24 h incubation at 30 °C, 3 ml aliquots of cell culture were spun down in 1.5 ml microcentrifuge tubes and the resultant cell pellets were visually inspected for EGFP expression. Ten EGFP-expressing colonies and five EGFP-negative colonies obtained using QuickStep-Cloning, and five EGFP-expressing colonies and five EGFP-negative colonies obtained using RF cloning [picked randomly from C41 (DE3) Kan + IPTG plate] were grown overnight at 37 °C in 5 ml 2 × TY media (16 g/l tryptone, 10 g/l yeast extract and 5 g/l NaCl). The recombinant plasmids were purified using QIAprep Spin Miniprep Kit (Qiagen) and sequenced by Source BioScience (Nottingham, UK). The same transformation protocol was used for *rfp* cloning experiment. The number of RFP-expressing colonies was determined by visual inspection. Three RFP-expressing colonies were used to inoculate: (i) 5 ml 2 × TY media, (ii) 5 ml 2 × TY media supplemented with 1 mM IPTG, and (iii) 5 ml 2 × TY media supplemented with 0.1 % w/v arabinose. After 48 h incubation at 30 °C, 3 ml aliquots of cell culture were spun down in 1.5 ml microcentrifuge tubes and the resultant cell pellets were visually inspected for RFP expression. Plasmids from five RFP-expressing colonies and five RFP-negative colonies obtained using QuickStep-Cloning were isolated and sent for sequencing.

## Additional file

Additional file 1:
**Estimated cloning times reported in Table 3 – calculations.** **Figure S1.** Plasmid map of pEGFP vector. **Figure S2.** Outline of *egfp* gene cloning experiment. **Figure S3.** Photograph of *E. coli *C41 (DE3) colonies in *egfp *cloning experiment. **Figure S4.** Photograph of cell pellets from cell cultures grown as part of *egfp *cloning experiment. **Figure S5.** Yield of whole plasmid amplification for different primer ratios used during asymmetric PCRs. **Figure S6.** Plasmid map of pBbA8k-RFP vector. **Figure S7.** Photograph of *E. coli *C41 (DE3) colonies in *rfp *cloning experiment. **Figures S8 and S9.** Photographs of cell pellets from cell cultures grown as part of *rfp *cloning experiment. (PDF 794 kb)

## Abbreviations

ABI-REC: Asymmetric bridge PCR with intramolecular homologous recombination
EGFP: Enhanced green fluorescent protein
EMP: Exponential megaprimer PCR
dsDNA: Double-stranded DNA
IFPC: Inverse fusion PCR cloning
LIC: Ligation Independent Cloning
PCR: Polymerase chain reaction
RAM: Recombination-assisted megaprimer
RF: Restriction-free
RFP: Red fluorescent protein
ssDNA: Single-stranded DNA

## References

[1] Tee KL, Wong TS (2013). Polishing the craft of genetic diversity creation in directed evolution. Biotechnol Adv.

[2] Lu Q (2005). Seamless cloning and gene fusion. Trends Biotechnol.

[3] Court DL, Sawitzke JA, Thomason LC (2002). Genetic engineering using homologous recombination. Annu Rev Genet.

[4] Zhu D, Zhong X, Tan R, Chen L, Huang G, Li J (2010). High-throughput cloning of human liver complete open reading frames using homologous recombination in Escherichia coli. Anal Biochem.

[5] Blanusa M, Schenk A, Sadeghi H, Marienhagen J, Schwaneberg U (2010). Phosphorothioate-based ligase-independent gene cloning (PLICing): An enzyme-free and sequence-independent cloning method. Anal Biochem.

[6] Shinomiya K, Mori T, Aoyama Y, Sera T (2011). Unidirectional cloning by cleaving heterogeneous sites with a single sandwiched zinc finger nuclease. Biochem Biophys Res Commun.

[7] Chen GJ, Qiu N, Karrer C, Caspers P, Page MG (2000). Restriction site-free insertion of PCR products directionally into vectors. Biotechniques.

[8] van den Ent F, Lowe J (2006). RF cloning: a restriction-free method for inserting target genes into plasmids. J Biochem Biophys Methods.

[9] Bryksin AV, Matsumura I (2010). Overlap extension PCR cloning: a simple and reliable way to create recombinant plasmids. Biotechniques.

[10] Miyazaki K (2011). MEGAWHOP cloning: a method of creating random mutagenesis libraries via megaprimer PCR of whole plasmids. Methods Enzymol.

[11] Papworth C, Bauer J, Braman J, Wright DA (1996). Site-directed mutagenesis in one day with >80 % efficiency. Strategies.

[12] Bi Y, Qiao X, Hua Z, Zhang L, Liu X, Li L (2012). An asymmetric PCR-based, reliable and rapid single-tube native DNA engineering strategy. BMC Biotechnol.

[13] Mathieu J, Alvarez E, Alvarez PJJ (2014). Recombination-assisted megaprimer (RAM) cloning. MethodsX.

[14] Ulrich A, Andersen KR, Schwartz TU (2012). Exponential megapriming PCR (EMP) cloning--seamless DNA insertion into any target plasmid without sequence constraints. PLoS One.

[15] Spiliotis M (2012). Inverse fusion PCR cloning. PLoS One.

[16] Wang BL, Jiao YL, Li XX, Zheng F, Liang H, Sun ZY (2009). A universal method for directional cloning of PCR products based on asymmetric PCR. Biotechnol Appl Biochem.

[17] Inoue H, Nojima H, Okayama H (1990). High efficiency transformation of Escherichia coli with plasmids. Gene.

[18] Gibson DG, Young L, Chuang RY, Venter JC, Hutchison CA, Smith HO (2009). Enzymatic assembly of DNA molecules up to several hundred kilobases. Nat Methods.

[19] Gibson DG (2011). Enzymatic assembly of overlapping DNA fragments. Methods Enzymol.

[20] Aslanidis C, de Jong PJ (1990). Ligation-independent cloning of PCR products (LIC-PCR). Nucleic Acids Res.

[21] Hanahan D (1983). Studies on transformation of Escherichia coli with plasmids. J Mol Biol.

