# Supplementary material for: QuickStep-Cloning: a sequence-independent, ligation-free method for rapid construction of recombinant plasmids
Source: J Biol Eng. 2015 Sep 18;9:15. doi: 10.1186/s13036-015-0010-3 (PMC4574722; doi:10.1186/s13036-015-0010-3)
Supplement: Additional file 1: — Estimated cloning times reported in Table 3 – calculations. Figure S1. Plasmid map of pEGFP vector. Figure S2. Outline of egfp gene cloning experiment. Figure S3. Photograph of E. coli C41 (DE3) colonies in egfp cloning experiment. Figure S4. Photograph of cell pellets from cell cultures grown as part of egfp cloning experiment. Figure S5. Yield of whole plasmid amplification for different primer ratios used during asymmetric PCRs. Figure S6. Plasmid map of pBbA8k-RFP vector. Figure S7. Photograph of E. coli C41 (DE3) colonies in rfp cloning experiment. Figures S8 and S9. Photographs of cell pellets from cell cultures grown as part of rfp cloning experiment. (PDF 794 kb) [file 13036_2015_10_MOESM1_ESM.pdf]

QuickStep-Cloning: a sequence-independent, ligation-free method for rapid construction of recombinant plasmids

Additional documentation

Pawel Jajesniak and Tuck Seng Wong

ChELSI Institute and Advanced Biomanufacturing Centre, Department of Chemical and Biological Engineering, University of Sheffield, Mappin Street, Sheffield S1 3JD, England

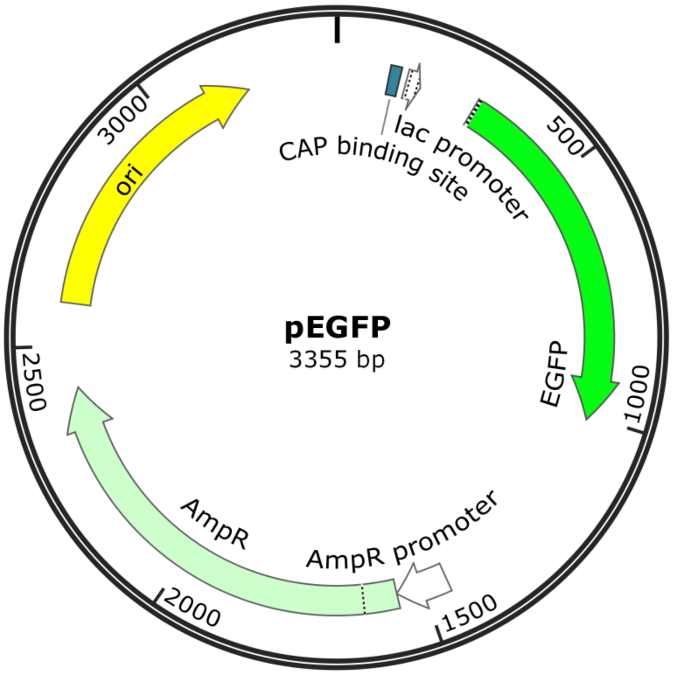

Suppl. Fig. S1. Plasmid map of pEGFP vector used in *egfp* cloning experiment (created with SnapGene).

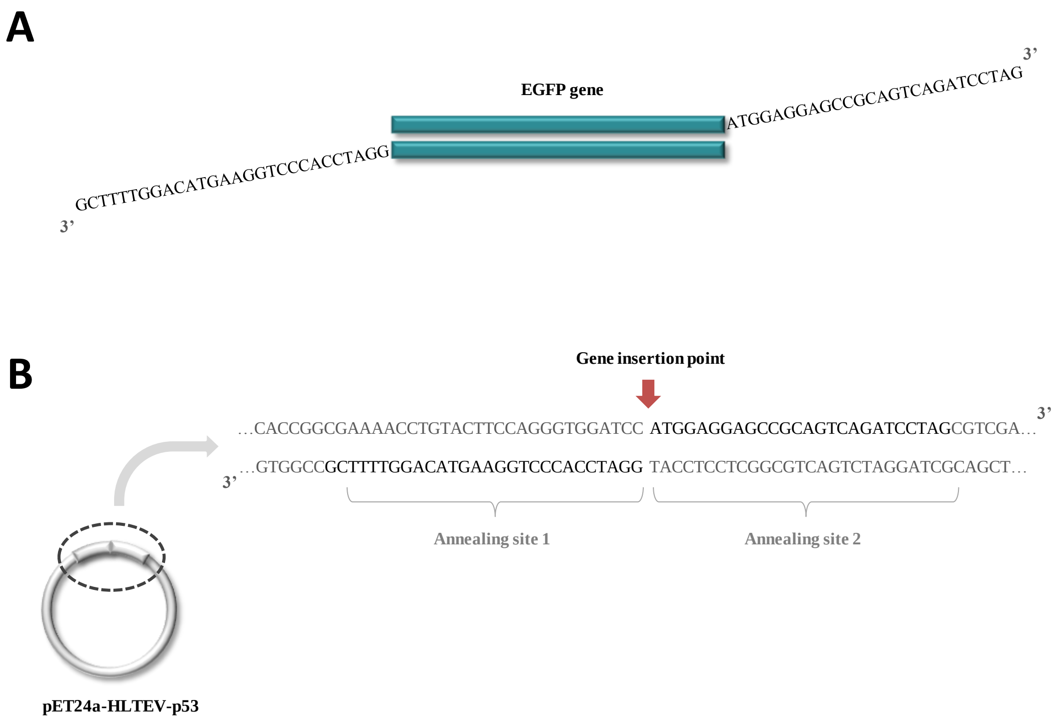

Suppl. Fig. S2. Outline of *egfp* gene cloning experiment. (A) Product of two asymmetric PCRs – *egfp* gene with 3' overhangs corresponding to the annealing sites flanking DNA insertion point present in pET24a-HLTEV-p53. (B) Sequence of the two megaprimer annealing sites.

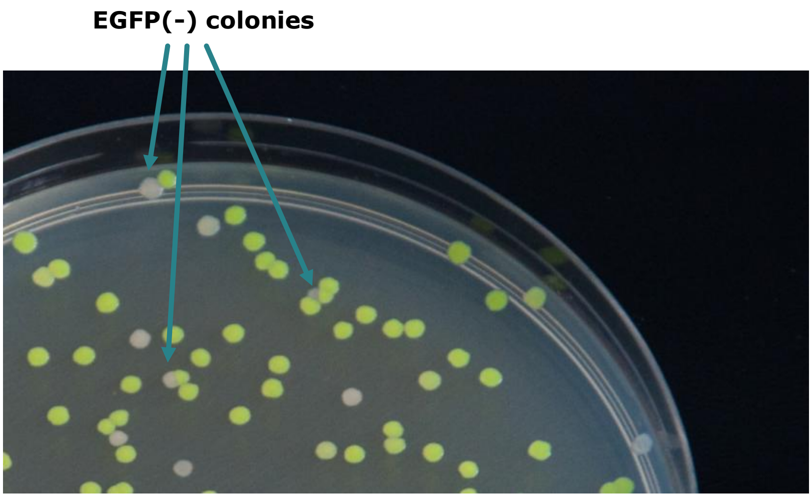

Suppl. Fig. S3. Photograph of *E. coli* C41 (DE3) colonies formed on agar plates supplemented with 50 µg/ml kanamycin and 1 mM IPTG after being transformed with the product of QuickStep-Cloning (as part of *egfp* cloning experiment). EGFP-expressing colonies are easily discernible even under visible light.

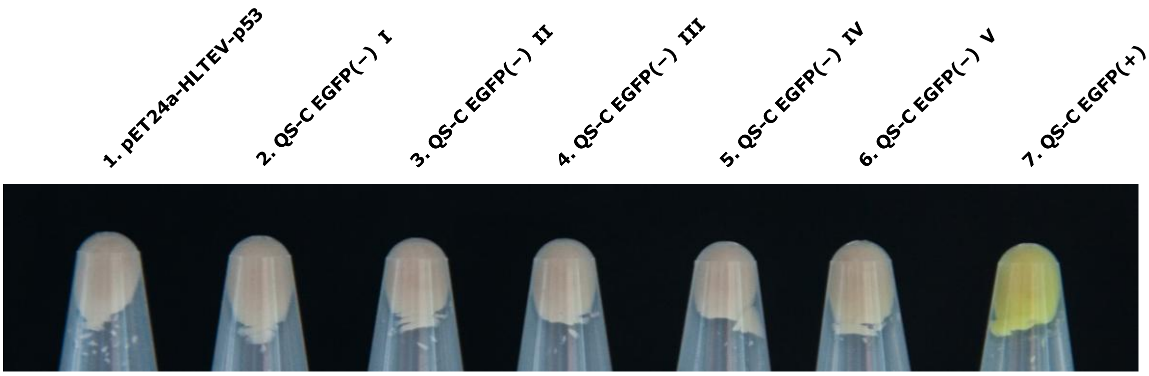

Suppl. Fig. S4. Cell pellets from cell cultures grown as part of *egfp* cloning experiment. Five EGFP-negative colonies together with one EGFP-expressing colony and one colony containing original pET24a-HLTEV-p53 were picked randomly and used to inoculate separate 5 ml aliquots of TB-based auto-induction media. After 24 h incubation at 30°C, 3 ml aliquots of cell culture were spun down and the resultant cell pellets were visually inspected for EGFP expression.

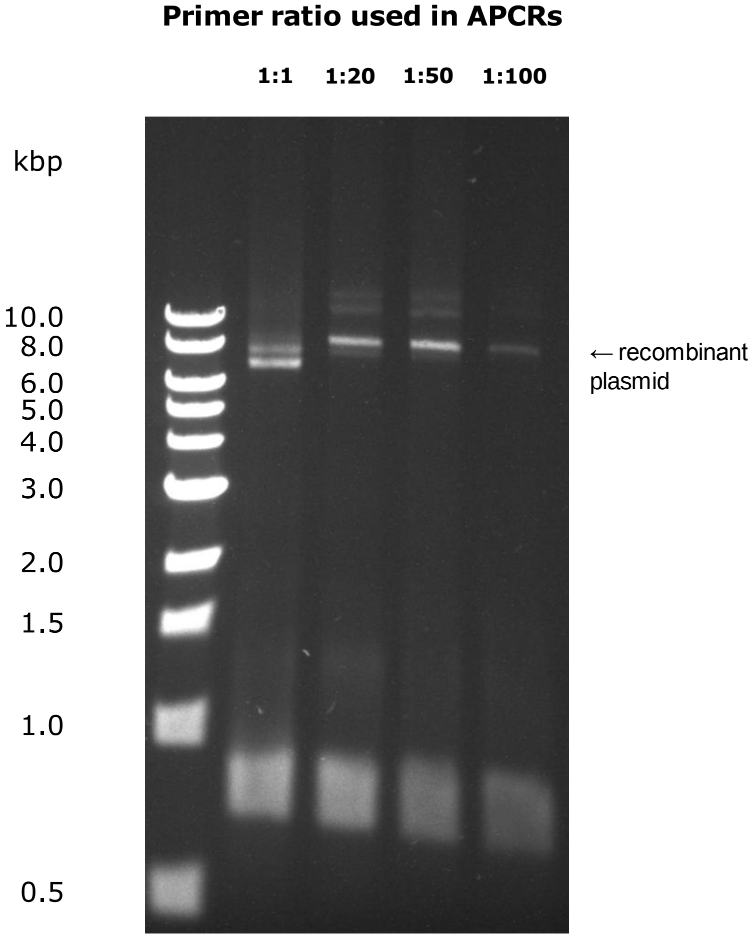

Suppl. Fig. S5. Yield of whole plasmid amplification for different primer ratios used during asymmetric PCRs.

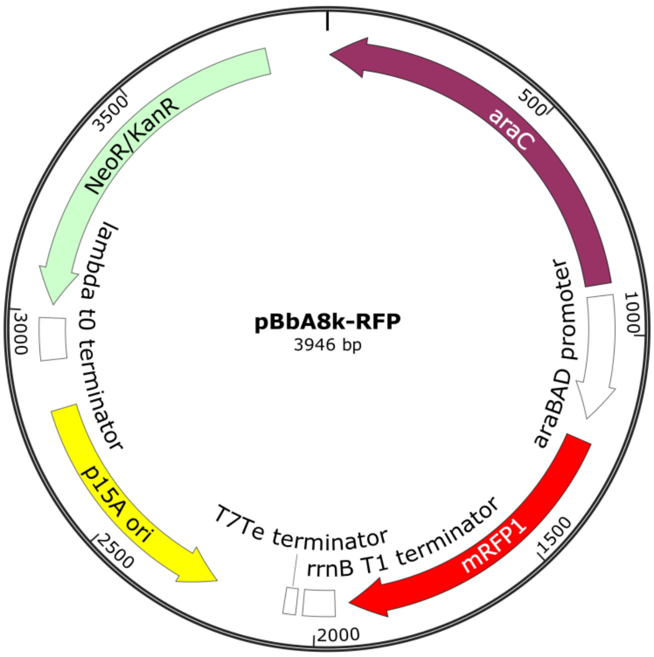

Suppl. Fig. S6. Plasmid map of pBbA8k-RFP vector used in *rfp* cloning experiment (created with SnapGene).

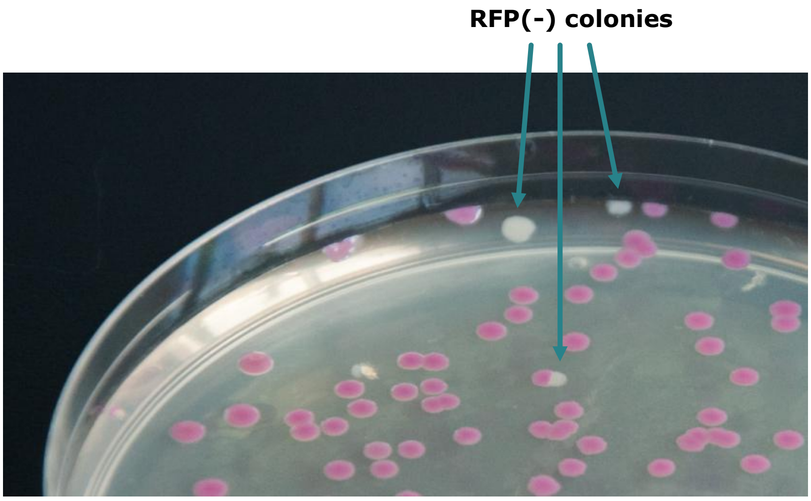

Suppl. Fig. S7. Photograph of *E. coli* C41 (DE3) colonies formed on agar plates supplemented with 50 µg/ml kanamycin and 1 mM IPTG after being transformed with the product of QuickStep-Cloning (as part of *rfp* cloning experiment). RFP-expressing colonies are easily discernible under visible light.

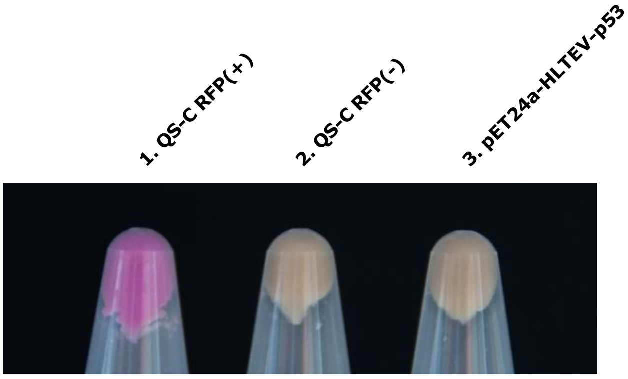

Suppl. Fig. S8. Cell pellets from cell cultures grown as part of *rfp* cloning experiment. One RFP-expressing colony together with one RFP-negative colony and one colony containing original pET24a-HLTEV-p53 were picked randomly and used to inoculate separate 5 ml aliquots of 2×TY media supplemented with 1 mM IPTG. After 48 h incubation at 30°C, 3 ml aliquots of cell culture were spun down and the resultant cell pellets were visually inspected for RFP expression.

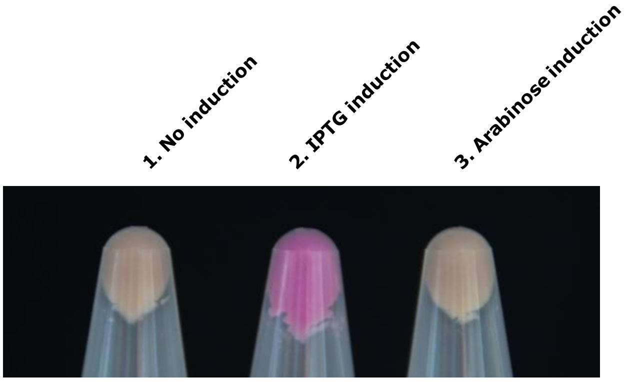

Suppl. Fig. S9. Cell pellets from cell cultures grown as part of *rfp* cloning experiment. Three RFP-expressing colonies were picked randomly and used to inoculate separate 5 ml aliquots of 2×TY media. One sample contained media only (1), one was supplemented with 1 mM IPTG (2) and 0.1% w/v arabinose was added to the remaining one (3). After 48 h incubation at 30°C, 3 ml aliquots of cell culture were spun down and the resultant cell pellets were visually inspected for RFP expression.

Estimated cloning times reported in Table 3 – calculations

Cloning times have been determined for the experiment involving insertion of 1 kb DNA fragment into 7 kb recipient plasmid. Time needed to prepare necessary PCR mixtures has not been included in the calculations (as it is very difficult to be accurately estimated). Exact times of particular steps involved in the PCR and recommended time of DpnI digestion and enzymatic phosphorylation-ligation have been sourced from references provided in Table 3. As a large fraction of PCR duration involves changing the temperature of reaction mixtures, additional 45 s has been added to each cycle to account for this fact – *e.g.*, estimated time of one cycle involving 7 s denaturation, 20 s annealing and 30 s extension is 102 s (57 s of incubation + 45 s needed for thermocycling).

1. QuickStep-Cloning

1st PCR:	30 cycles: 7 s denaturation, 20 s annealing, 30 s/kb extension<br>Final extension: -<br>Total PCR duration time for 1 kb insert: 1 h
Purification:	Column-based PCR purification<br>Estimated time: 30 min
2nd PCR:	25 cycles; 10 s denaturation, 20 s annealing, 30 s/kb extension<br>Final extension: 2 min<br>Total PCR duration time for 7 kb recipient plasmid: 2 h
DpnI digestion	15 min
Phosphorylation-ligation	None
Transformation	1 h 30 min
**Total time**	**5 h 15 min**

2. RF cloning

1st PCR:	20 cycles: 7 s denaturation, 20 s annealing, 30 s/kb extension<br>Final extension: -<br>Total PCR duration time for 1 kb insert: 40 min
Purification:	Column-based PCR purification<br>Estimated time: 30 min
2nd PCR:	35 cycles; 30 s denaturation, 60 s annealing, 120 s/kb extension<br>Final extension: -<br>Total PCR duration time for 7 kb recipient plasmid: 9 h 30 min
DpnI digestion	2 h
Phosphorylation-ligation	None
Transformation	1 h 30 min
**Total time**	**14 h**

3. ABI-REC

1st PCR:	30 cycles: 15 s denaturation, 30 s annealing, 60 s/kb extension<br>Final extension: 2 min<br>Total time for 1 kb insert and 7 kb recipient plasmid: 4 h 15 min
Purification:	None
2nd PCR:	None
DpnI digestion	2 h
Phosphorylation-ligation	None
Transformation	1 h 30 min
**Total time**	**7 h 45 min**

4. RAM cloning

1st PCR:	20 cycles: 35 s denaturation, 35 s annealing, 30 s/kb extension<br>Final extension: 5 min<br>Total PCR duration time for 1 kb insert: 1 h
Purification:	Gel extraction<br>Estimated time: 1h 30 min
2nd PCR:	15 cycles; 35 s denaturation, 35 s annealing, 30 s/kb extension<br>Final extension: 5 min<br>Total PCR duration time for 7 kb recipient plasmid: 1 h 30 min
DpnI digestion	2 h 20 min
Phosphorylation-ligation	None
Transformation	1 h 30 min
**Total time**	**7 h 45 min**

5. EMP cloning

1st PCR:	25 cycles: 10 s denaturation, 30 s annealing, 15 s/kb extension<br>Final extension: -<br>Total PCR duration time for 1 kb insert: 45 min
Purification:	Column-based PCR purification<br>Estimated time: 30 min
2nd PCR:	25 cycles; 10 s denaturation, 30 s annealing, 30 s/kb extension<br>Final extension: 2 min<br>Total PCR duration time for 7 kb recipient plasmid: 2 h
DpnI digestion	30 min
Phosphorylation-ligation	2 h
Transformation	1 h 30 min
**Total time**	**7 h 15 min**

5. IFPC

1st PCR:	25 cycles: 15 s denaturation, 20 s annealing, 30 s/kb extension
	Final extension: 7 min
	Total PCR duration time for 1 kb insert: 1 h
Purification:	Gel extraction
	Estimated time: 1h 30 min
2nd PCR:	25 cycles; 20 s denaturation, 30 s annealing, 30 s/kb extension
	Final extension: 7 min
	Total PCR duration time for 7 kb recipient plasmid: 2 h
DpnI digestion	None
Phosphorylation-ligation	30 min
Transformation	1 h 30 min
**Total time**	**6 h 30 min**
